# Delayed diagnosis adversely affects outcome in systemic lupus erythematosus: Cross sectional analysis of the LuLa cohort

**DOI:** 10.1177/0961203320983445

**Published:** 2021-01-05

**Authors:** Anna Kernder, Jutta G Richter, Rebecca Fischer-Betz, Borgi Winkler-Rohlfing, Ralph Brinks, Martin Aringer, Matthias Schneider, Gamal Chehab

**Affiliations:** 1 Department of Rheumatology and Hiller-Research Unit Rheumatology, Heinrich-Heine-University, Düsseldorf, Germany; 2 German Lupus Self-Help Community, Wuppertal, Germany; 3 Department of Medicine III, Division of Rheumatology, TU Dresden, Germany

**Keywords:** Outcome, delay, diagnosis, SLE, Lupus

## Abstract

**Objective:**

Despite increased physician’s awareness and improved diagnostic and serological testing in the recent years, the interval between the initial symptoms and the diagnosis of Systemic lupus erythematosus (SLE) is still very long. Our aim was to study this delay and its association to the outcome of the disease.

**Methods:**

Information on demographics, onset of first symptoms, first physicians visit and time of diagnosis was assessed by self-reported questionnaires among SLE patients in Germany (LuLa cohort, n = 585) in the year 2012. Disease activity (Systemic Lupus Activity Questionnaire; SLAQ), disease related damage (Brief Index of Lupus Damage; BILD), health related quality of life (Short Form 12) and fatigue (FSS) were chosen as proxies for outcome. Linear regression analysis was used to analyze the association of the delay in diagnosis to the outcome, adjusted for age, disease duration and sex.

**Results:**

Mean duration between the onset of symptoms and the diagnosis of SLE was 47 months (SD 73). The longer the time to diagnosis, the higher the disease activity (β = 0.199, p < 0.0001), the disease-related damage (β = 0.137, p = 0.002) and fatigue (β 0.145, p = 0.003) and the lower the health-related quality of life (physical β = −0.136, p = 0.004, mental β = −0.143, p = 0.004).

**Conclusion:**

In systemic lupus erythematosus, longer time to diagnosis was associated with worse outcome. Concepts in care with the intention to shorten the time to diagnosis are needed to improve the long-term outcome of the disease.

## Introduction

The initial symptoms of systemic lupus erythematosus are often nonspecific and mimic other medical conditions, increasing the risks for diagnostic delay.^1^ The heterogeneity of possible manifestations makes early diagnosis and subsequent disease management more difficult and can delay effective treatment. In a study of 121 SLE patients in the UK, 70% of the participants stated that they had initially received another diagnosis. A median of ten consultations with three different doctors were required before a diagnosis was finally made.^2^ Despite of increased physician’s awareness and improved diagnostic and serological testing in the recent years, the interval between the onset of the first symptoms and the diagnosis of SLE is still very long. For example, Ozbek et al. reported a mean delay of 21.8 ± 30.3 months in 136 Turkish SLE patients in 2003, with arthralgia being the most common symptom (60%) at the time of diagnosis and Sawah et al. described a delay of 67.2 ± 87.5 months in 2015 in a US-cohort (n = 827).^3^

Reducing this delay may enable monitoring and treatment at an earlier stage before severe organ involvement might have occurred. In a Danish cohort of 100 patients with lupus nephritis followed for 15 years, a delayed diagnosis and intervention increased the risk of progression to end-stage renal disease (ESRD).^4^ Furthermore, an US health insurance database study reported that the diagnosis of SLE being delayed for more than 6 months from symptom onset leads to greater health care utilization, flare rates, and more insurance claims in the following years.^5^

In addition, both the path to diagnosis and the diagnosis itself imply a wide range of stressors, limitations, fears and uncertainties for patients, which can affect all areas of their lives.^6,7^ As a result, their participation und health related quality of life are often severely and permanently impaired.

Even though the impact of time to diagnosis on the development of ESRD, the frequency of flares and the frequency of physician’s visits have been described, the association to the quality of life, overall disease damage and disease activity has yet not been investigated. This information is crucial to understand the potential impact of an early diagnosis and to improve the multidisciplinary management in practice.

## Methods

### Data source

The LuLa study is a nationwide survey among SLE patients that was established in the year 2001. SLE-patients receive a questionnaire every year, asking about demographic data, clinical parameters such as comorbidities, lupus-specific medication, disease activity, damage and health-related quality of life.^8^ Data from year 2012 was analysed in this study. In 2012 we additionally inquired about the time to diagnosis and the organ involvement at the time of diagnosis.

Study organization and preparation of data acquisition were performed by the German SLE patient association (GSPA), the Lupus erythematodes Selbsthilfegemeinschaft. Pseudonymized data collection and scientific evaluation were guaranteed by our tertiary center. Independent of the study, medical care for the included patients is provided by physicians all over Germany. The study organization and implementation was chosen to minimize the effect of an expectancy bias, such as the Rosenthal effect.^9^

Participants were enrolled by invitation of their rheumatologist or the GSPA itself. The inclusion criteria for the study were a confirmed diagnosis of SLE and the returning of the completed questionnaire.

To reduce data entry errors for the digitization of the questionnaires two-pass verification was performed at the tertiary center.

In comparison with the reference data from the national database of the German Rheumatism Research Center, it was shown previously that data collected by LuLa study is reliable, comparable and can be considered as representative of SLE patients in Germany.^8^

The questionnaire was sent to 636 patients by the GSHC in the year 2012, the return rate of the completed questionnaires was 91.2% (n = 585).

### Outcome

We chose disease activity, disease-related damage and health-related quality of life, assessed by patient reported questionnaires, as outcome parameters. We are not able to record death as an outcome parameter in our cohort as it is a patient-reported survey.

To assess disease activity, the patient reported Systemic Lupus Activity Questionnaire (SLAQ),^10^ was used, which was translated and validated in different languages. The questionnaire uses 24 items to capture disease symptoms in the previous 3 months. The German version shows a strong correlation with the physician reported systemic lupus activity measure (SLAM) and presents good to excellent internal consistency.^11^

The patient reported Brief Index of Lupus Questionnaire (BILD)^12^ was used for assessing disease-related damage. It consists of 28 items enquiring about organ damage accumulated since the diagnosis of SLE. It was likewise validated in different languages. The German version has proven a comparable validity to the original BILD and a strong correlation with the physician-reported damage score (SDI).^13^

The Short Form 12 Health Survey (SF-12)^14^ was used to assess the health-related quality of life (HRQoL). Based on the Short Form 36 (SF-36), the SF-12 provides comparable results with a mental (MCS) and a physical (PCS) component. Additionally, the physical functioning index of the SF-36 (SF-36-pfi) was assessed.^15^

Fatigue was evaluated by the Fatigue Severity Scale (FSS) measuring the impact of fatigue on nine specific types of functioning in the previous two weeks. A score of < 4 is considered to be normal.^16^

### Time to diagnosis and statistical analysis

**Figure 1.**
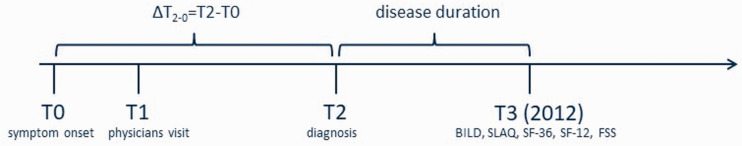
Timeline representing the requested time points. T0: onset of symptoms, T1: first physicians visit, T2: diagnosis of SLE, T3: time of the survey (2012): asking about T0, T1, T2 and the outcome of the disease by self-reported questionnaires (BILD, SLAQ, SF-36, SF-12, FSS).

The interval between the onset of symptoms, the first physician’s visit and the time of diagnosis (Figure 1) as well as the organ involvement at the time of diagnosis was recorded.

Using linear regression we analyzed the association of the interval between the onset of symptoms and the time of diagnosis to the outcome of the disease. The analysis was adjusted for age, sex, disease duration und organ involvement at the time of diagnosis. T-test was used to compare delays and outcome in two different groups of patients.

Data was analyzed with the statistical software program R (The R Foundation for statistical computing, Vienna, Austria).

The LuLa study was approved by the Heinrich-Heine-University Duesseldorf institutional review board (study numbers 2260 and 3708) and is registered in the German World Health Organization primary registry ‘German Clinical Trial Register’, www.germanctr.de (ID: DRKS00011053). The study complies with the Declaration of Helsinki. The study did not require additional approval.

## Results

### Time to diagnosis

In total, 585 patients were included in our analysis with a mean age of 53.3 (SD 12.2) years and mean disease duration of 17.7 (SD 7.9) years.

Mean time to diagnosis (ΔT_2-0_) was 47 months, including 13 months from first symptoms to the first physician’s visit and 34 months from the first physicians visit to the diagnosis SLE (Table 1, Figure 1).

**Table 1.** Characteristics of the study cohort in the year 2012. 585 patients participated. Lupus medication includes NSAIDs, steroids, antimalarials, azathioprine, methotrexate, leflunomide, ciclosporine A, mycophenolic acid, cyclophosphamide, rituximab and belimumab.

	% (n)	Mean (SD)	Median (IQR)	Range
Female	94.4 (552)			
Age		53.3 (12.3, )	50 (17)	14–87
Disease duration (years)		17.7 (7.9)	16 (11)	7–51
Symptoms to first physicians visit (month)		13.2 (40.9)	2 (5)	0–336
Physicians visit to diagnosis (month)		34.0 (62.7)	8 (36)	0–576
Symptoms to diagnosis (month)		47.2 (72.6)	13 (56)	0–576
Physical functioning (SF 36 PFI)		67.0 (28.6)	75 (45)	0–100
Physical quality of life (SF-12 PCS)		40.1 (12.0)	41.1 (20.8)	11.6–64.4
Mental quality of life (SF-12 MCS)		46.4 (11.4)	49.6 (17.8)	16.3–68.2
Disease activity (SLAQ)		13.0 (7.9)	11 (11)	0–42
Damage (BILD)		2.5 (2.4)	2 (3)	0–12
Fatigue (FSS)		4.1 (2.0)	4.4 (3.4)	1–7
Number of comorbidities		1.7 (1.2)	0 (2)	0–10
Number of lupus medication		1.8 (1.1)	2 (2)	0–5
Number of other medication		2.6 (1.7)	3 (2)	0–8
Prednisolone ≤7,5 mg/d	50.5 (294)			
Prednisolone > 7,5 mg/d	12.5 (73)			

**Figure 2.**
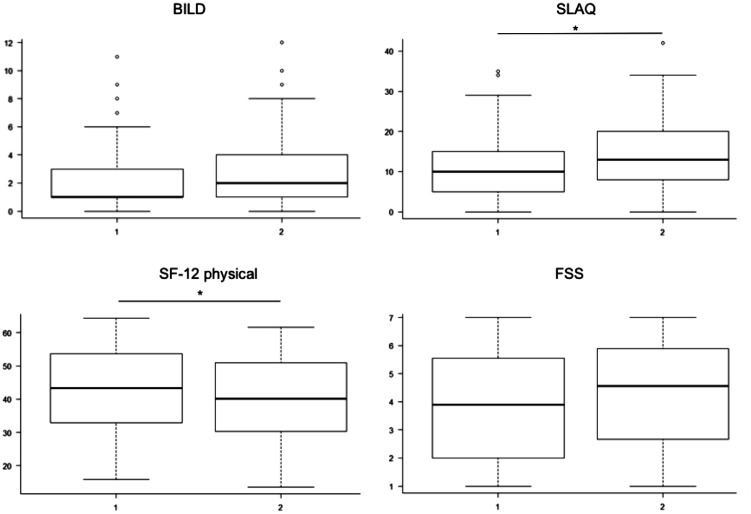
Effects of time to diagnosis on disease-related damage (BILD), disease activity (SLAQ), health related quality of life (SF-12) and fatigue in the year 2012. Boxplots presenting two groups of patients: 1 presenting patients that reported a time to diagnosis of less than 6 month from the onset of symptoms (n = 264), 2 presenting patients that reported a time of more than 6 month (n = 321). T-Test for comparison of the two groups. *p < 0.05.

40.3% of our patients reported skin involvement (n = 236) and 40.5% joint involvement (arthritis and arthralgia) at the time of diagnosis (T2), whereas lung (7.7%), heart (6.8%), kidneys (13.3%) were affected less often at the time of diagnosis. We found evidence that patients with cerebral and mental involvement at onset (neuropsychiatric SLE (NPSLE), n = 76) had longer delays in diagnosis. In detail, patients with NPSLE reported a mean time to diagnosis of 69 months, compared to 43 months in patients having an organ involvement other than NPSLE at the time of diagnosis (p = 0.018, T-test). This delay was mainly due to the time between the first physician visit and diagnosis (T2-T1, Figure 2). Patients with NPSLE reported a delay of 56 months vs. 31 months that were reported by patients with another manifestation (p = 0.001, T-test). No differences were reported in the time between the onset of symptoms (T0) and the first physician’s visit (T2), (15 months vs. 13 months, p = 0.740, T-Test). Patients with joint involvement at the time of diagnosis also reported a longer time to diagnosis (53.0 month) compared to patient having an organ involvement other than joints at the time of diagnosis (39.8 month), although this did not reached statistical significance (p = 0.051). Details are given in Table 2.

**Table 2.** Organ involvement at the time of diagnosis. A Percentage of organ involvement at the time of diagnosis of the total population (n = 585) B Mean time between the onset of symptoms and the diagnosis of SLE divided by the organ involvement at the time of diagnosis (Yes = organ involved at the time of diagnosis, No = organ not involved at the time of diagnosis). Significant differences were seen in NPSLE (neuropsychiatric SLE), (T-test, *p < 0.05).

A			B		
	%	n	Yes	No	p-value
Kidney	13.3	78	44.5	46.0	0.300
Heart	6.8	40	50.1	45.4	0.664
Skin	40.3	236	42.5	49.9	0.250
Joints	40.5	237	53.0	39.8	0.051
Lung	7.7	45	58.9	44.5	0.516
NPSLE *	13.1	76	68.7	42,5	0.018*
Musculoskeletal	5.0 (29)	29	62.1	43.8	0.098

### Time to diagnosis predicts outcome

Linear regression analysis adjusted for disease duration, sex and age was used to analyze the association between the time to diagnosis and the outcome of the disease. Disease activity (SLAQ), disease related damage (BILD), mental and physical HRQoL (SF-12), physical functioning (SF-36 PFI) and fatigue (FSS) in the year 2012 were chosen as proxies for the outcome of the disease.

Linear regression analysis revealed: the longer the time to diagnosis, the higher the disease-related damage (β 0.137, p = 0.002), the higher the disease activity (β 0.199, p < 0.0001) and lower the health-related quality of life (SF-12 physical β −0.136, p = 0.004, SF-12 mental β −0.143, p = 0.004) in 2012. Additionally, fatigue was rated higher (β 0.145, p = 0.003). Table 3 shows more details (R^2^ and standard errors). A sensitivity analysis including organ involvement at time of diagnosis did not change the results of the regression model.

**Table 3.** Time to diagnosis predicts outcome. Linear regression adjusted for sex, age and disease duration. Independent variable: Duration between onset of symptoms and diagnosis. Dependent variable: outcome parameters (BILD, SLAQ, SF-12 mental/physical and SF-36-PFI, FSS). β stand. regression coefficient, HRQoL Health related quality of life. *p < 0.05, **p < 0.01; ***p < 0.001.

Dep. variable	adj. R^2^	β_stand._	SD	p-value
Disease related damage				
BILD **	0.163	0.137	3.119	0.0019
Disease activity				
SLAQ ***	0.068	0.199	4.276	<0.0001
HRQoL				
SF-12 mental **	0.012	−0.143	−2.882	0.0042
SF-12 physical **	0.125	−0.136	−2.897	0.0040
Physical functioning index				
SF-36 PFI *	0.152	−0.088	−1.997	0.0465
Fatigue				
FSS **	0.018	0.145	3.0046	0.0025

Furthermore, we compared two groups of patients. Group 1 includes patients with a time to diagnosis of less than 6 months (n = 264) and group 2 includes patients with more than 6 months to diagnosis (n = 321), Figure 2. Patients significantly differed in SLAQ (median (range)) (11(0–42) vs. 14(0–34), p = 0.0001), SF-12 physical (42.4 (15.8–64.4) vs. 35.6 (11.6–62.3), p = 0.09), SF-36 pfi (75 (0–100) vs. 65 (0–100), p = 0.02), but not in BILD (2.4(0–11) vs. 2.9 (0–11), p = 0.05), SF-12 mental (49.9 (19.0–68.2) vs. 48.2 (16.2–65.0), p = 0.52), and FSS (4.1(1–7) vs. 4.4(1–7), p = 0.07) in 2012 (≤6 months and > 6 months respectively, T-test).

## Discussion

SLE significantly impairs the life of those affected. In our cohort, the health related quality of life, for example, was strongly impaired, comparable to patients with progressed cancer in the last 6–12 month of their life.^17^ In addition to the impact of disease activity, accumulated damage caused by the disease itself and side effects of the treatment contributes to the deterioration of quality of life.^18^ Thus a delayed diagnosis, the uncertainty caused by a delayed diagnosis and the resulting delay in therapy may be of importance.^3^ Therefore, we analysed the impact of a delayed diagnosis on long-term outcome.

Our data show a median delay of 47 months from the first SLE symptom to the diagnosis of SLE, comparable with the reported data of an US-cohort of 827 patients from Sawah et al.^3^ Interestingly, the time to diagnosis in both cohorts was mainly due to the time between the first physician’s visit and diagnosis (34 ± 61.7 in our cohort vs. 41 ± 64.8 months in the cohort of Sawah et al.). In both countries, the mean time from the onset of symptoms to the first physician’s visit (13 months in our cohort versus 25 months in the US-cohort) was significantly lower compared to the time between the first physician’s visit and diagnosis. This underlines the challenge of diagnosing the disease SLE.

The marked difference of 12 months (13 vs. 25 months) for the time to the first physician consultation in the two cohorts may be due to differences in the health care systems in these countries. In Germany, the threshold for consulting a physician may be lower due to the statutory health insurance system. Therefore, the indicated duration of 13 months is rather surprisingly long. As we did not record the first healthcare provider to whom patients addressed their initial symptoms, we cannot assess the impact of poorer availability of specialist care (e.g. rheumatologists) to the reported delay.

The long time between the first physician’s visit and diagnosis indicates that clinical factors (e.g. detection and classification of symptoms, diagnostics) most often contributed to the delay. Since we cannot provide information on the treating health care professional in charge, it is not possible to attribute this problem to a specific sector of care (primary, secondary or tertiary care). Therefore, we can only assume that training and awareness campaigns in primary care as well as optimised access routes to rheumatologists or national and international reference centres could possibly contribute to improved and faster diagnosis.

This idea is underlined by a comparison of our data with the data of Ozbek et al. who observed a shorter delay in diagnosis of ‘only’ 21.8 months.^19^ The data of the research group were obtained from 136 patients who were diagnosed at a tertiary centre with specialized units for Rheumatology and Nephrology. In contrast, our data and the data of Sawah et al. include patients being diagnosed not selectively by a tertiary centre but by all levels of health care (also primary and secondary care, for instance general practitioners or municipal hospitals). We know from our cohort that more than 30% are primarily cared for by a non-rheumatologist even in long-term care.^20^

Our data shows even more pronounced delays among patients with NPSLE at the time of diagnosis (n = 76) than in SLE patients with other manifestations. This delay was also mainly caused by the time between the first physician’s visit and diagnosis. Psychological and mental health symptoms were previously reported in the Lupus UK’s on line survey to be associated with delayed diagnosis and initial misdiagnosis.^21,22^

These findings should prompt clinical review and consideration of further investigation in patients with unclear neurological or psychiatric symptoms. As mentioned in the EULAR recommendations for the management of systemic lupus erythematosus with neuropsychiatric manifestation (2010), NPSLE continues to pose diagnostic and therapeutic challenges to practising physicians. The diagnostic work-up is time-consuming, including examination of the cerebrospinal fluid, Electroencephalography (EEG), blood tests, neuroimaging, as well as tests for neuropsychological and cognitive dysfunction,^23^ and usually requires an interdisciplinary assessment by a neurologist or psychiatrist, which additionally contributes to delay in diagnoses.

### Patient-reported data

By study design, all of our data is patient-reported. Patients were asked to have a firm diagnosis of SLE ideally confirmed in written by their physician. The used patient-oriented questionnaires for disease activity (SLAQ), disease related damage (BILD) and health-related quality of life (SF-12/SF-36) have shown high correlation with physician-reported information.^11,13–15^ Related to the study design we were not able to precisely assess the clinical situation at disease onset and its influence to the time to diagnosis (e.g. due to lack of serological markers or physician-reported-outcomes). Thus a confirmation of the influence of diagnostic delay on supplemental outcome parameter and laboratory findings reported by physicians at disease onset would further strengthen our findings.

There remains some uncertainty whether the initial symptoms reported by the patients were attributable to the later diagnosed disease SLE or whether they were assigned to another disease. In addition, there is the possibility of a bias by outcome that cannot be excluded. Patients with poor outcomes may estimate the interval between the first symptoms and the time of diagnosis to be longer compared to patients with a better outcome at the time of the interview.^24^

Furthermore, patients with long disease may have difficulties in remembering the dates of the first symptoms and the diagnosis (recall bias). To minimize this impact, the regression model was adjusted for disease duration.

### Time to diagnosis predicts outcome in SLE

Our linear regression analysis revealed that delays in diagnosis were associated with lower health related quality of life and higher disease related damage, fatigue and disease activity in 2012.

Of course, we cannot exclude confounding factors that influence the outcome since the diagnosis of SLE, such as the quality of medical care.^20^

The effect of a prolonged time to diagnosis on outcome may, for instance, be related to sustained disease activity promoting the development of damage in the absence of adequate therapy for an undiagnosed disease. As we know from the study from Bruce et al., diagnostic delay in SLE favours an accelerated accumulation of damage, which in turn leads to a decrease in quality of life, fatigue and increased mortality.^25^ This association is also known from the treatment of other rheumatic diseases like rheumatoid arthritis and psoriatic arthritis where a delayed diagnosis and start of therapy are known to accelerate damage and consequently functional impairment.^26,27^

In general, one we would rather expect a faster diagnosis in patients with severe organ manifestation or high disease activity. Since we only had data on organ involvement, but not on the severity of the disease at the time of diagnosis, we were unable to investigate this association any further.

In addition to the impact of an early diagnosis and start of a therapy on the physical outcome of the disease, it has been shown for other diseases, such as adrenal insufficiency, that early diagnosis also improves the psychiatric health status.^28^ Conversely, inadequate coping or catastrophising due to a long undiagnosed but limiting disease can be associated with increased anxiety and depression (important aspects of QoL), which are closely related to fatigue.^29,30^

## Conclusion

Our study shows links between the time to diagnosis and important SLE outcome parameter such as quality of life, disease-related damage and disease activity, assessed by self-reported questionnaires. An early diagnosis could therefore be a good approach to improve the outcome of patients with systemic lupus erythematosus. Patients with NPSLE reported the longest time to diagnosis in our cohort. These findings should prompt clinical review and consideration of further investigation in patients with unclear neurological or psychiatric symptoms.

Training and awareness campaigns in primary care, optimised access routes to rheumatologists as well as accelerated access to specialized rheumatology centers may contribute to an early diagnosis and a better outcome in consequence.

## References

[1] Rees F Doherty M Lanyon P, et al. Early clinical features in systemic lupus erythematosus: can they be used to achieve earlier diagnosis? A risk prediction model. Arthritis Care Res (Hoboken) 2017; 69: 833–841. 27588834 10.1002/acr.23021

[2] Kent T Davidson A Newman D Buck G D’Cruz D. Burden of illness in systemic lupus erythematosus: results from a UK patient and carer online survey. Lupus 2017; 26: 1095–1100. 28406053 10.1177/0961203317698594

[3] Al Sawah S Daly RP Foster S, et al. SAT0423 understanding delay in diagnosis, access to care and satisfaction with care in Lupus: Findings from a cross-sectional online survey in the United States. Ann Rheum Dis 2015; 74: 812.3–812.

[4] Faurschou M Dreyer L Kamper A-L Starklint H Jacobsen S. Long-term mortality and renal outcome in a cohort of 100 patients with lupus nephritis. Arthritis Care Res 2010; 62: 873–880. 10.1002/acr.20116 20191478

[5] Oglesby A Korves C Laliberté F, et al. Impact of early versus late systemic lupus erythematosus diagnosis on clinical and economic outcomes. Appl Health Econ Health Policy 2014; 12: 179–190. 24573911 10.1007/s40258-014-0085-x

[6] Chehab G Carnarius H Schneider M. What matters for lupus patients? Presse Medicale Paris Fr 1983. Presse Med 2014; 43: e197–e207. 24791601 10.1016/j.lpm.2014.03.004

[7] Robinson D Aguilar D Schoenwetter M, et al. Impact of systemic lupus erythematosus on health, family, and work: the patient perspective. Arthritis Care Res (Hoboken) 2010; 62: 266–273. 20191527 10.1002/acr.20077

[8] Fischer-Betz R Wessel E Richter J Winkler-Rohlfing B Willers R Schneider M. [Lupus in Germany: analysis within the German lupus self-help organization (LULA).]. Z Rheumatol 2005; 64: 111–122. 15793677 10.1007/s00393-005-0644-5

[9] Rosenthal R Fode K. The effect of experimenter bias on the performance of the albino rat. Behav Sci 2007; 17: 8:183–189.

[10] Karlson EW Daltroy LH Rivest C, et al. Validation of a Systemic Lupus Activity Questionnaire (SLAQ) for population studies. Lupus 2003; 12: 280–286. 12729051 10.1191/0961203303lu332oa

[11] Chehab G Richter J Sander O Fischer-Betz R Ostendorf B Al-Neyadi T. Validation and evaluation of the German version of the Systemic Lupus Activity Questionnaire (SLAQ). Clin Exp Rheumatol 2015; 33: 354–359. 25797042

[12] Yazdany J Trupin L Gansky SA, et al. Brief index of lupus damage: a patient-reported measure of damage in systemic lupus erythematosus. Arthritis Care Res (Hoboken) 2011; 63: 1170–1177. 21584946 10.1002/acr.20503 PMC3149719

[13] Chehab G Sander O Richter J, et al. Validation and evaluation of the German Brief Index of Lupus Damage (BILD)–a self-reported instrument to record damage in systemic lupus erythematosus. Lupus 2013; 22: 1050–1055. 23963433 10.1177/0961203313500369

[14] Ware J Kosinski M Keller SD. A 12-Item Short-Form Health Survey: construction of scales and preliminary tests of reliability and validity. Med Care 1996; 34: 220–233. 8628042 10.1097/00005650-199603000-00003

[15] Ware J. SF-36 health survey: manual and interpretation guide. Boston: New England Medical Center, 1993.

[16] Krupp LB LaRocca NG Muir-Nash J Steinberg AD. The fatigue severity scale. Application to patients with multiple sclerosis and systemic lupus erythematosus. Arch Neurol 1989; 46: 1121–1123. 2803071 10.1001/archneur.1989.00520460115022

[17] Raijmakers NJH Zijlstra M van Roij J Husson O Oerlemans S van de Poll-Franse LV. Health-related quality of life among cancer patients in their last year of life: results from the PROFILES registry. Support Care Cancer Off J Multinatl Assoc Support Care Cancer. 2018; 26: 3397–3404. 10.1007/s00520-018-4181-6 29663137

[18] Poomsalood N Narongroeknawin P Chaiamnuay S Asavatanabodee P Pakchotanon R. Prolonged clinical remission and low disease activity statuses are associated with better quality of life in systemic lupus erythematosus. Lupus 2019; 28: 1189–1196. 31307256 10.1177/0961203319862614

[19] Ozbek S Sert M Paydas S Soy M. Delay in the diagnosis of SLE: the importance of arthritis/arthralgia as the initial symptom. Acta Med Okayama 2003; 57: 187–190. 14627070 10.18926/AMO/32807

[20] Kernder A Richter JG Fischer-Betz R, et al. Quality of care predicts outcome in systemic lupus erythematosus: a cross-sectional analysis of a German long-term study (LuLa cohort). Lupus 2020; 29: 136–143. 31992161 10.1177/0961203319896626 PMC6993135

[21] Sloan M Harwood R Sutton S, et al. Medically explained symptoms: a mixed methods study of diagnostic, symptom and support experiences of patients with lupus and related systemic autoimmune diseases. Rheumatol Adv Pract 2020; 4: rkaa006. 32373774 10.1093/rap/rkaa006 PMC7197794

[22] Morgan C Bland AR Maker C Dunnage J Bruce IN. Individuals living with lupus: findings from the LUPUS UK Members Survey 2014. Lupus 2018; 27: 681–687. 29310537 10.1177/0961203317749746 PMC5888773

[23] Bertsias G Ioannidis JPA Boletis J, et al. EULAR recommendations for the management of systemic lupus erythematosus. Report of a Task Force of the EULAR Standing Committee for International Clinical Studies Including Therapeutics. Ann Rheum Dis 2008; 67: 195–205. 17504841 10.1136/ard.2007.070367

[24] Baron J Hershey JC. Outcome bias in decision evaluation. J Pers Soc Psychol 1988; 54: 569–579. 3367280 10.1037//0022-3514.54.4.569

[25] Bruce IN O'Keeffe AG Farewell V, et al. Factors associated with damage accrual in patients with systemic lupus erythematosus: results from the Systemic Lupus International Collaborating Clinics (SLICC) Inception Cohort. Ann Rheum Dis 2015; 74: 1706–1713. 24834926 10.1136/annrheumdis-2013-205171 PMC4552899

[26] Lard LR Visser H Speyer I, et al. Early versus delayed treatment in patients with recent-onset rheumatoid arthritis: comparison of two cohorts who received different treatment strategies. Am J Med 2001; 111: 446–451. 11690569 10.1016/s0002-9343(01)00872-5

[27] Haroon M Gallagher P FitzGerald O. Diagnostic delay of more than 6 months contributes to poor radiographic and functional outcome in psoriatic arthritis. Ann Rheum Dis 2015; 74: 1045–1050. 24525911 10.1136/annrheumdis-2013-204858

[28] Bleicken B Ventz M Quinkler M Hahner S. Delayed diagnosis of adrenal insufficiency is common: a cross-sectional study in 216 patients. Am J Med Sci 2010; 339: 525–531. 20400889 10.1097/MAJ.0b013e3181db6b7a

[29] Fischin J Chehab G Richter JG, et al. Factors associated with pain coping and catastrophising in patients with systemic lupus erythematosus: a cross-sectional study of the LuLa-cohort. Lupus Sci Med 2015; 2: e000113–e000113. 26629351 10.1136/lupus-2015-000113 PMC4654099

[30] Arnaud L Gavand PE Voll R, et al. Predictors of fatigue and severe fatigue in a large international cohort of patients with systemic lupus erythematosus and a systematic review of the literature. Rheumatol Oxf Engl 2019; 58: 987–996. 10.1093/rheumatology/key398 30597077

